# Labeling Extracellular Vesicles for Nanoscale Flow Cytometry

**DOI:** 10.1038/s41598-017-01731-2

**Published:** 2017-05-12

**Authors:** Aizea Morales-Kastresana, Bill Telford, Thomas A. Musich, Katherine McKinnon, Cassandra Clayborne, Zach Braig, Ari Rosner, Thorsten Demberg, Dionysios C. Watson, Tatiana S. Karpova, Gordon J. Freeman, Rosemarie H. DeKruyff, George N. Pavlakis, Masaki Terabe, Marjorie Robert-Guroff, Jay A. Berzofsky, Jennifer C. Jones

**Affiliations:** 1 Molecular Immunogenetics and Vaccine Research Section, Vaccine Branch, CCR, NCI, NIH, Bethesda, MD USA; 2 Experimental Transplantation and Immunology Branch, CCR, NCI, NIH, Bethesda, MD USA; 3 Immune Biology of Retroviral Infection Section, Vaccine Branch, CCR, NCI, NIH, Bethesda, MD USA; 4 Vaccine Branch Flow Core Facility, CCR, NCI, NIH, Bethesda, MD USA; 5 Human Retrovirus Section, Vaccine Branch, CCR, NCI, NIH, Bethesda, MD USA; 6 Optical Microscopy Core, LRGBE, CCR, NCI, NIH, Bethesda, MD USA; 7 Dana-Farber Cancer Institute, Boston, MA USA; 8 Stanford University School of Medicine, Stanford, CA USA

## Abstract

Extracellular vesicles (EVs), including exosomes and microvesicles, are 30–800 nm vesicles that are released by most cell types, as biological packages for intercellular communication. Their importance in cancer and inflammation makes EVs and their cargo promising biomarkers of disease and cell-free therapeutic agents. Emerging high-resolution cytometric methods have created a pressing need for efficient fluorescent labeling procedures to visualize and detect EVs. Suitable labels must be bright enough for one EV to be detected without the generation of label-associated artifacts. To identify a strategy that robustly labels individual EVs, we used nanoFACS, a high-resolution flow cytometric method that utilizes light scattering and fluorescence parameters along with sample enumeration, to evaluate various labels. Specifically, we compared lipid-, protein-, and RNA-based staining methods and developed a robust EV staining strategy, with the amine-reactive fluorescent label, 5-(and-6)-Carboxyfluorescein Diacetate Succinimidyl Ester, and size exclusion chromatography to remove unconjugated label. By combining nanoFACS measurements of light scattering and fluorescence, we evaluated the sensitivity and specificity of EV labeling assays in a manner that has not been described for other EV detection methods. Efficient characterization of EVs by nanoFACS paves the way towards further study of EVs and their roles in health and disease.

## Introduction

The characterization of individual Extracellular Vesicles (EVs) is challenging due to the small size of EVs^1^. Bulk methods of EV analysis, such as quantitative PCR, western blots and mass spectrometry^1, 2^, are assays of the general population in a sample, rather than assays of individual EVs or distinct EV subsets. Flow cytometric analyses of bead-bound EVs permit the enrichment of specific EV populations of interest by using antibodies that capture EVs for bulk analysis but without multiparametric information at the single-EV level^3–5^. Electron Microscopy^6^, Nanoparticle Tracking Analysis (NTA)^7^, Tunable Resistive Pulse Sensing^8^ and nanofluidics^9–11^ methods are useful to characterize the size and concentration of EVs in a solution. However, these methods cannot assess the complex profiles of subsets of EVs^12^ with multiple labels evaluated for each EV in the manner that we use cytometric methods to analyze multiple labels on individual cells, to identify various types and subsets.

**Figure 1:**
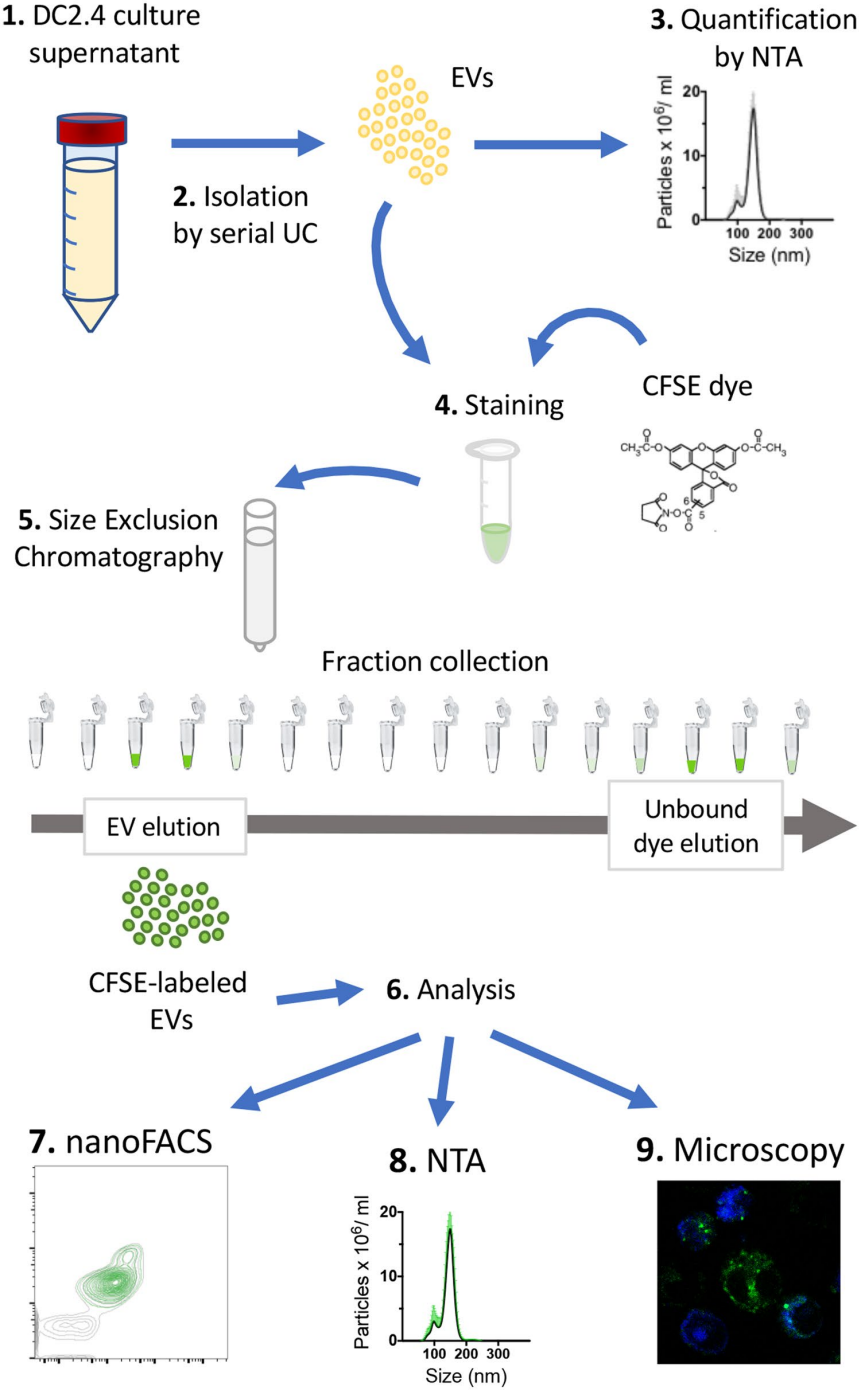
Summary of the workflow for the methods described in this manuscript. DC2.4 cells were cultured in EV-depleted medium without phenol red to produce EV containing supernatants (1). Then, EVs were isolated by serial ultracentrifugation^31^ (2) and concentration and size distribution characterized by NTA (3). Afterwards, EVs were stained with CFSE (4) or other dyes (not depicted here) and free dye was washed by size exclusion chromatography (5). CFSE-labeled EVs eluted in fractions 3 and 4 were used for their analysis (6) by different methods: nanoFACS (7), NTA (8) and microscopy (9). UC, ultracentrifugation; EV, extracellular vesicle; NTA, Nanoparticle Tracking Analysis.

Two major limiting factors are the limits of detection of the instruments being used and the presence of artifacts that arise during sample collection and processing. Therefore, we developed nanoFACS, a high resolution flow cytometry (HR-FCM) method for analyzing and sorting individual EVs and other nanoscale particles (e.g. liposomal products, HIV). NanoFACS uses high sensitivity multiparametric scattered light and fluorescence measurements, in contrast to many HR-FCM methods that rely on fluorescent triggering with bulk EV labels^13–17^. The multiparametric capabilities of nanoFACS enabled us to comprehensively evaluate the performance of various labeling methods with unprecedented detail and precision. Specifically, this manuscript presents how we use the nanoFACS method to (1) detect background levels of unbound labels and (2) evaluate different labeling methods, and thereby identify a method that generates fewer background contaminants during the labeling process. Herein we describe the use of nanoFACS to resolve unlabeled EVs and report an inexpensive and efficient strategy for staining single EVs brightly and uniformly, while maximizing the EV fluorescence signal to background reference noise ratio and keeping functional EV properties active, as summarized in Fig. 1.

## Results and Discussion

### Analysis of EVs with nanoFACS

**Figure 2:**
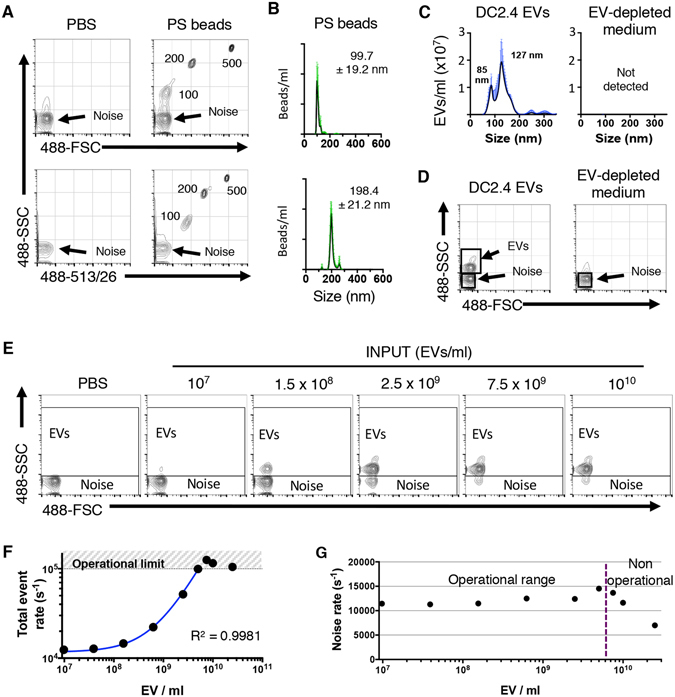
Submicron particle detection by nanoFACS HR-FCM. (**A**) Representative dot plot of PBS or 100, 200 and 500 nm polystyrene beads, analyzed by light scattering and fluorescence by nanoFACS, with background reference noise shown in the lower left corner in each plot. The background reference noise is a random sampling of scattered light from laser:stream intercept. (**B**) Size distribution of 100 and 200 nm polystyrene beads and (**C**) DC2.4-derived EVs (left) or control sample from EV-depleted medium subjected to same isolation procedure (right) by NTA. (**D**) DC2.4 EV detection by light scattering in nanoFACS, clearly resolved above the background reference noise. (**E**) Serially diluted DC2.4 EV analisis by nanoFACS to assess the suitable operational range that avoids coincident detection of particles. The relative percentage of noise and EVs particles changes as the EV concentration increases, but the light scattering pattern doesn’t change. (**F**) Quantification of the total event rate in **E**. Dotted line depicts the limit of the operational range. The curve fit was calculated by nonlinear regression excluding the three most concentrated EV preparations. (**G**) Noise rate is stable in the operational range, but drops when sample concentration is above the operational range (gate strategy shown in **E**). Representative data from multiple independent experiments with similar results. NTA histograms represent the mean of three independent acquisitions ± SD in green. The numbers on the NTA graphs indicate the mode value of the size. EV, extracellular vesicle; Noise, background reference noise; FSC, forward and SSC, side light scatter; NTA, Nanoparticle Tracking Analyses; SD, standard deviation.

The sensitivity of nanoFACS was demonstrated with fluorescent polystyrene beads (Fig. 2A,B). 100 nm beads could be easily resolved above the background instrument noise (hereafter referred to as background reference noise), both by light scattering and fluorescence (Fig. 2A). EVs isolated and purified from the culture supernatant of immature dendritic cells (DC2.4 cell line), but not control EV-depleted medium, revealed a homogeneous 126.7 ± 4 nm population by Nanoparticle Tracking Analysis (NTA) (Fig. 2C) consistent with exosomes^1^. Analysis of DC2.4 EVs with nanoFACS by light scatter demonstrated complete resolution of the EV population from background reference noise (Fig. 2D). To confirm that individual EV analysis with nanoFACS was robust, without swarming or coincident event detection^14^, we serially diluted DC2.4 EVs in PBS, and determined an operational range where the event rate increase was proportional to the EV concentration, with stable signal intensities, confirming no coincident detection in the system with event rates below 100,000/second (Fig. 2E–G).

### Detection of micelles or aggregates of commonly used amphiphilic labels

**Figure 3:**
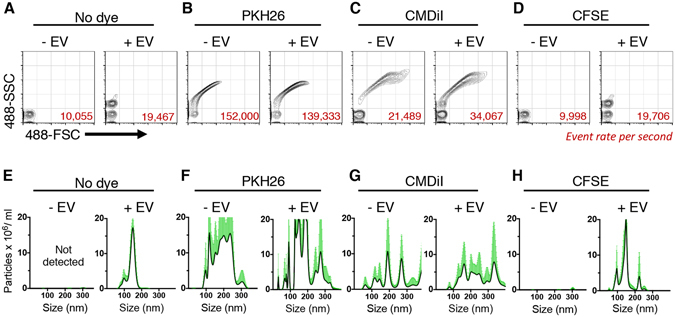
EV label suitability analysis by nanoFACS HR-FCM and NTA. nanoFACS analysis of light scattering pattern and event rate (**A–D**), and assessment of concentration and size distribution by NTA (**E–H**) of the following samples: PBS control and unstained EVs diluted in PBS (**A**,**E**), PKH26 alone in PBS or in the presence of EVs (**B**,**F**), CM-DiI alone in PBS or with EVs (**C**,**G**) and CFSE alone in PBS or with EVs (**D**,**H**). Side by side comparisson between PBS control and dye alone was used to interrogate the non-specific formation of micelles or other forms of dye aggregation. All the samples were stained and tested on the same day, under the same nanoFACS and NTA setup conditions to avoid interexperimental variations. Representative data from three independent experiments is shown. NTA histograms represent the mean of three replicate meassurements of the same sample and SD in green. EV, extracellular vesicle; FSC, forward and SSC, side light scatter; NTA, Nanoparticle Tracking Analyses.

We used nanoFACS to test the suitability of different dyes to stain EVs in bulk for efficient identification of EVs with a general label. Using DC2.4-derived EVs stained with PKH26, a widely used amphiphilic lipid dye^18, 19^, the light scattering pattern of stained EVs was different from unstained EVs (Fig. 3A,B). We also observed a higher event rate in the PKH26-stained EV sample (red numbers in Fig. 3), which indicates an increase of the particle concentration, which was confirmed with NTA (Fig. 3E,F). PKH26 dye alone in PBS, when combined with the PKH diluent buffers, without added EVs, demonstrated a similar polydisperse particle distribution, consistent with the presence of 100–400 nm micelles or aggregates of PKH26. CM-DiI, a structurally similar to PKH26 but water-soluble dye, also produced non-specific aggregates in the presence of EVs or alone in solution (Fig. 3C), which was further confirmed by NTA analysis (Fig. 3G). We also tested the dye SYTO RNASelect, which binds to nucleic acids, but found vesicle/dye aggregation without successful staining (data not shown).

### Assessment of amine-reactive dye, CFSE, as an EV label

As lipid and RNA dyes produced artifacts, we tested the protein-binding dye 5-(and-6)-Carboxyfluorescein Diacetate Succinimidyl Ester or CFDA-SE (hereinafter referred to as CFSE)^20, 21^. CFSE alone did not form aggregates and CFSE-stained EVs maintained a similar light scattering pattern to unstained EVs (Fig. 3A and D). Moreover, nanoFACS event rate and NTA analysis of concentration and size distribution of particles in CFSE-stained EV sample resembled to that observed in unstained EV sample (Fig. 2E,H). We used spike in beads to quantitatively analyze the concentration of particles following each labeling method and confirmed that staining EVs with CFSE, as opposed to lipid binding dyes, did not create artifactual aggregates (Supplementary Fig. 1). Thus, both nanoFACS and NTA data confirm that CFSE does not undergo non-specific aggregation, in contrast to lipid and RNA-binding dyes, and is suitable to stain single EVs for nanoscale flow cytometry for analytical and quantitative purposes.

### Use of size exclusion chromatography to reduce background fluorescence

**Figure 4:**
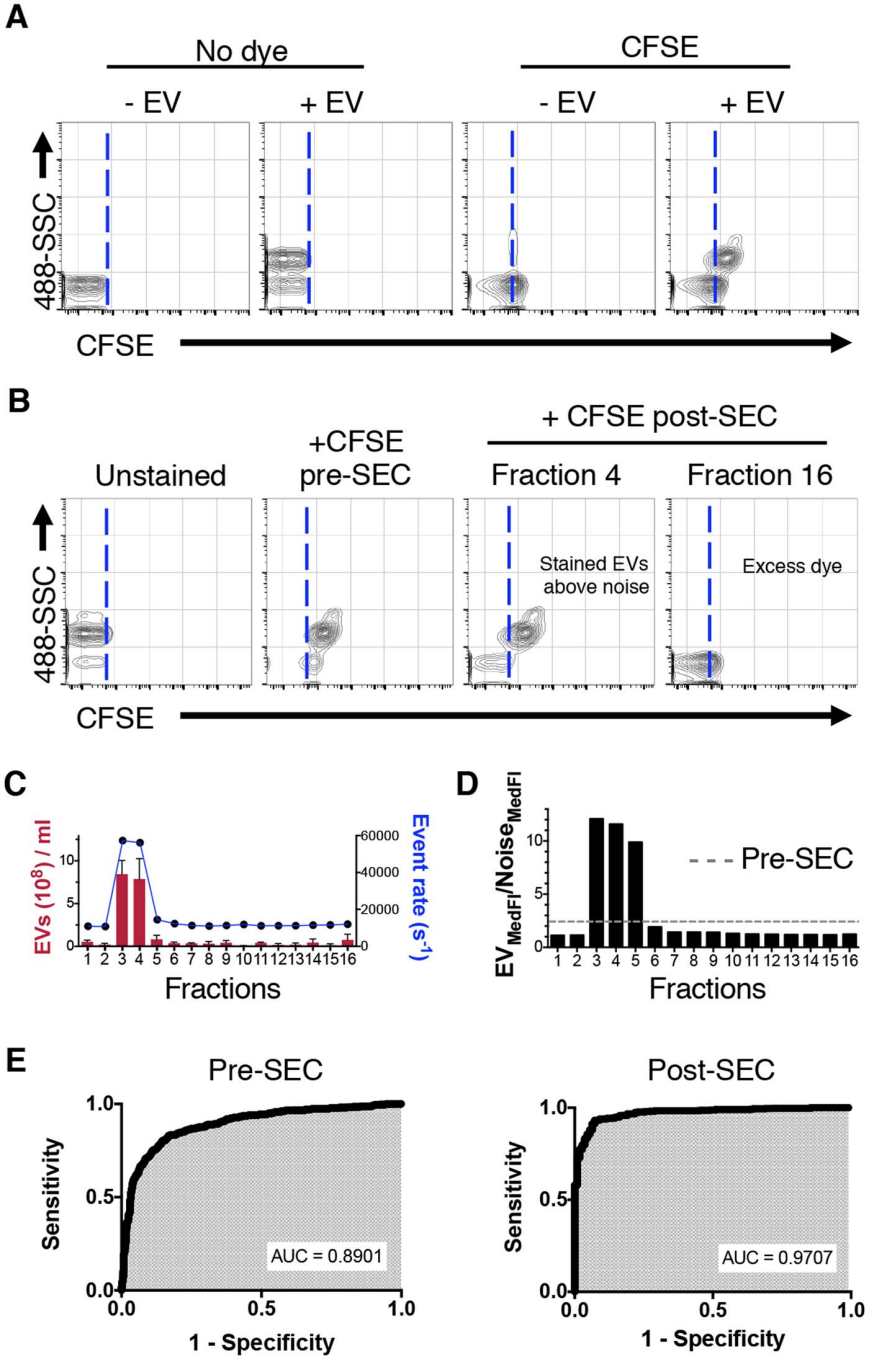
Size exclusion chromatography removal of unbound dye increases the signal to noise ratio of CFSE-labeled EVs. (**A**) Representative plots of fluorescence detection on unstained EVs, CFSE-labeled EVs and EV-lacking controls, analyzed by nanoFACS. Note a shift on the background reference noise fluorescence, due to the presence of free fluorescent CFSE dye in the sample^22, 23^. (**B**) Representative dot plots depicting 488-SSC and CFSE fluorescence showing unstained and CFSE-stained EVs before and after size exclusion chromatography. Dotted lines in **A** and **B** separate the CFSE^-^ and CFSE^+^ events, and were set on the limit of the background reference noise population in the unstained EV plot. (**C**) EV concentration analysis by NTA (in red) and total event rate by nanoFACS (in blue) of each fraction collected after size exclusion chromatography. NTA data shows the mean of three acquisitions and SD. (**D**) Ratio between EV and background reference noise median fluorescence intensity after eluting from size exclusion column (gated as in Fig. 2E). Dashed line shows the ratio before chromatography. Analysis of size exclusion chromatography fractions was performed twice with similar results. (**E**) ROC curves to assess specificity and sensitivity of EV identification by fluorescence. EV, extracellular vesicle; FSC, forward and SSC, side light scatter; MedFI, Median Fluorescence Intensity. SEC, size exclusion chromatography; ROC, Receiver Operating Characteristic; AUC, Area Under the Curve; FSC, forward scatter; SSC, side scatter.

Labeling with CFSE rendered EVs fluorescently detectable by nanoFACS, but also produced a shift of the fluorescence in the background reference noise events (Fig. 4A), as a result of free dye in the sample that spontaneously hydrolyze and turn the stream fluorescent^22, 23^. To remove unbound CFSE fluorescence, we found that size exclusion chromatography, which has been used for isolating EVs from plasma^24^, more effectively removed unbound label as compared to pelleting EVs by ultracentrifugation, sucrose cushions, or CFSE sequestration with BSA-coated beads (Fig. 4B and Supplementary Fig. 2). Monitoring the EV concentration as indicated by the event rate in nanoFACS and NTA analysis showed that over 90 or 78%, respectively, of the total EVs were collected in fractions 3 and 4 (Fig. 4C and Supplementary Fig. 2). Improved staining after NAP-5 size exclusion chromatography (Fig. 4D) was further ratified by enhanced sensitivity and specificity shown by area under the curve (AUC) and receiver operating characteristics (ROC, Fig. 4E) measurements. Based on the non-enzymatic kinetics of CFDA-SE/CFSE protein-binding and fluorescence^22, 23^, we optimized incubation times and incubation temperatures for EV labeling with CFSE. Longer incubation times improved EV staining, as measured by the EV_MedFI_/Noise_MedFI_ ratio. However, it also decreased EV concentration, in a time and temperature-dependent manner, as shown in the event rate with nanoFACS and EV concentration values by NTA (Supplementary Fig. 3), which may be attributable to EV rupture or adhesion to the tube during incubation.

### Assessment of EV integrity after size exclusion chromatography

**Figure 5:**
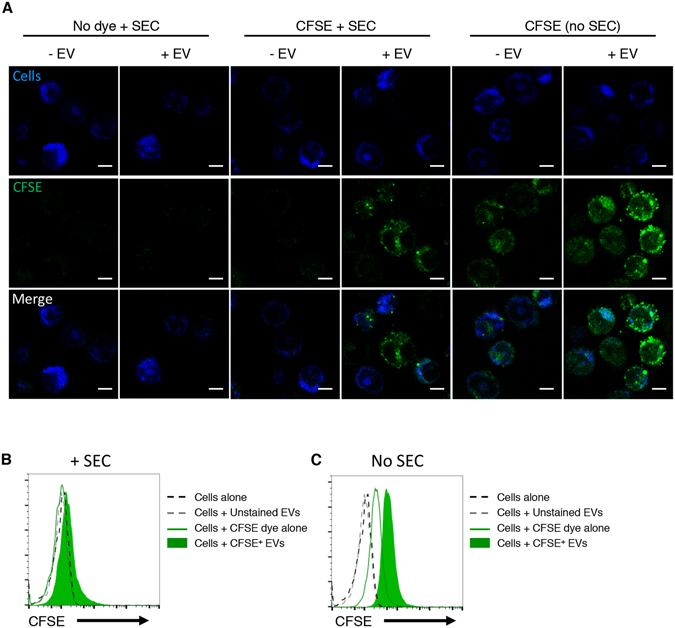
DC2.4 dendritic cells uptake CFSE-labeled EVs. (**A**) Confocal microscopy experiment showing that DC2.4 cells (in blue, stained with Cell Trace Far Red dye) uptake DC2.4 EVs labeled with CFSE (in green) following the staining and size exclusion chromatography method. After size exclusion chromatography, only cells incubated with CFSE labeled EVs are green. In the absence of size exclusion chromatography, cells are fluorescent regardless of the presence of EVs, indicating that unbound dye is the major contributor to the cell staining. Representative images of 5 analyzed fields are shown. All images were scaled in the same way. Scale bar = 5 µm. The experiment was repeated twice with similar results. (**B**) and (**C**) Fluorescence analysis by flow cytometry of cells shown in **A**, with (**B**) and without (**C**) size exclusion chromatography after EV labeling. The experiment was repeated three times, with similar results. EV, extracellular vesicle; SEC, size exclusion chromatography.

Several reports suggest that size exclusion chromatography preserves EV structural and functional integrity, more so than ultracentrifugation methods^25–28^. To confirm quantitative, morphological, and functional preservation of EV integrity with the labeling and chromatographic methods presented here, we performed NTA (Fig. 3H) and EV internalization studies^29^. The uptake of CFSE-labeled EVs is evident in the increased CFSE fluorescent signal on DC2.4 cells incubated with CFSE-labeled EVs, and is absent in cells incubated with unstained EVs or dye alone. Thus, CFSE labeling and subsequent washing of free dye by size exclusion chromatography does not interfere with the ability of CFSE-labeled EVs to be taken up by cells (Fig. 5A,B). The importance of removing unbound label was further demonstrated by the assessment of non-specific fluorescence of cells incubated with labeled EVs, with or without the SEC clean up step. Cells incubated with labeled EVs without SEC or with dye alone also acquired fluorescence, suggesting that the free dye was incorporated in the cells via direct binding of unbound CFSE to cellular proteins (Fig. 5A,C).

We repeated the experiment but labeling EVs with PKH26 dye instead and using PKH26 dye alone as a control. After incubating the cells with labeled EVs, we observed that the dye was incorporated in the cell membranes, even in the absence of EVs^30^ (Supplementary Fig. 4 **)**. This observation suggests that free PKH26 dye is not efficiently removed from the EV preparation, even after the ultracentrifugation step on a sucrose cushion^31^.

To demonstrate that CFSE-labeled EVs retain this functional capacity with molecular specificity, we tested whether CFSE-labeled EVs uptake is enhanced by TIM-4 expression (Supplementary Fig. 5). Previous studies demonstrated that TIM-4 expressing 3T3 cells take up phosphatidylserine-positive EVs via the interaction of cell surface TIM-4 with phosphatidylserine on EV surfaces^32, 33^.

### Quantitation of Single EV Fluorescence

**Figure 6:**
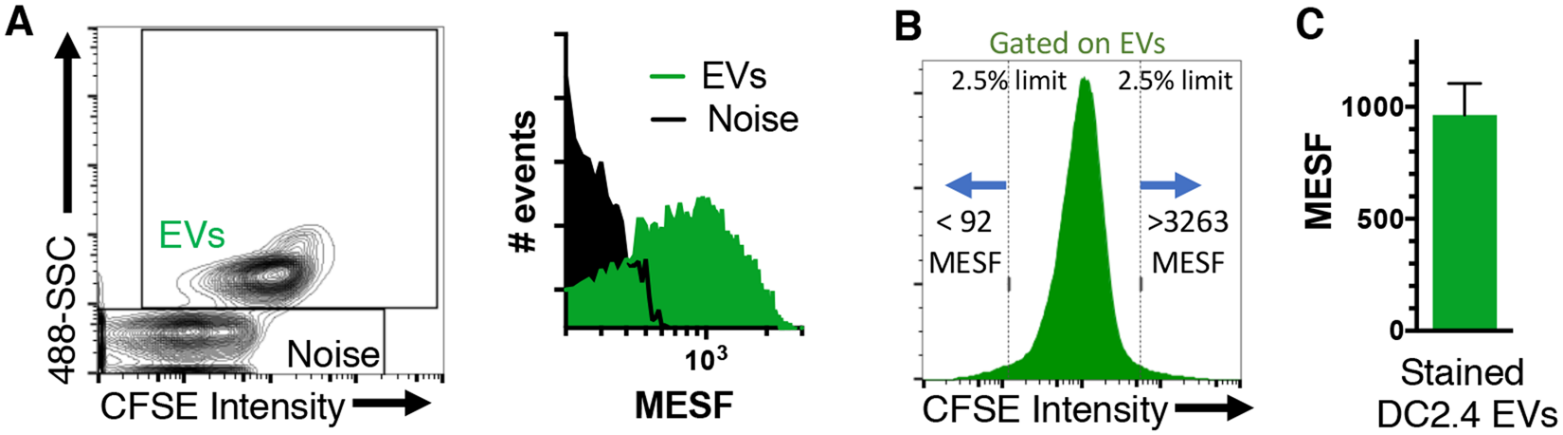
Fluorescence quantification of CFSE molecules on labeled EVs. (**A**) Background reference noise and EV fluorescence intensity transformed into a MESF histogram, see Supplementary Figure 6 for additional analysis. (**B**) Fluorescence detection range of the 95% of total EVs and (**C**) average MESF + SEM of EVs stained with CFSE, from four independent experiments. MESF, Molecules of Equivalent Soluble Fluorochrome.

Molecules of Equivalent Soluble Fluorochrome (MESF) beads were used to quantitate fluorescence based on nanoFACS fluorescence intensities^13^ (Fig. 6). DC2.4 EVs were stained with a range of 92 to 3,263 CFSE molecules, and a median average of 963 (Fig. 6B,C). The limit of detection varied depending on the criteria used to define the CFSE^+^ and CFSE^−^ populations (Supplementary Fig. 6). We also assessed the stability of the CFSE-labeled EVs upon different storage conditions and found that fluorescence was maintained at 4 °C or after freezing (Supplementary Fig. 7). Dual SSC and fluorescence analysis with nanoFACS was critical for EV identification and fluorescence quantification.

## Conclusions

Bulk analyses of heterogeneous EV populations have limited utility if distinct subpopulations of EVs carry different molecular repertoires, which correlate with specific EV sources and functions. With the rapid evolution of technologies for resolving EVs with cytometry, there is increasing need for robust labeling methods to clearly visualize individual EVs and discriminate EV subpopulations. Here we present the first comparison of HR-FCM staining protocols with the three main classes of general EV labels: lipid-binding, protein-binding, and nucleic acid-binding, for single EV resolution. The nanoFACS method used in this study enabled us to interrogate samples from both light scatter and fluorescence channels to characterize these labels more comprehensively than previously reported methods that use a fluorescent threshold/trigger setting^14–17^. NanoFACS detects artifactual shifts in the fluorescence of the background reference noise due to unbound labels and detects nanomaterial composition changes. Such changes and artifacts are not fully detected with previously published methods because relevant signals fall below the trigger level.

The EV labeling method with CFSE as optimized here is also suitable for other cytometric modalities to track EVs, such as microscopy, in addition to flow cytometry. Furthermore, removing unbound labels with size exclusion chromatography preserves biological function of EVs^25–28^, in a manner that can be compromised by other widely used methods, such as sucrose or Optiprep gradients, due to osmotic effects on the EVs^34^. The robust stability of fluorescence to freeze-thaw cycles makes these CFSE-labeled EVs potential candidates to use among different laboratories and as reference materials for the standardization of fluorescence in the HR-FCM field.

Overall, further development of nanoFACS and other high resolution cytometric methods, along with new bulk EV staining techniques, and high performance epitope-specific labels, will build upon the EV-staining and EV-analysis methods described here, and pave the road for characterization of specific EV subsets and subsequent studies of their roles *in vivo*.

## Methods

### Cell culture and EV production

The immature dendritic cell line DC2.4 was kindly provided by Kenneth Rock (University of Massachusetts Medical School, Boston, MA) and cultured in phenol red free RPMI1640 medium supplemented with 10% FBS, 1% L-Glutamine, 1% Penicillin-Streptomycin and 0.1% β-mercaptoethanol (ThermoFisher). 3T3 and 3T3-mTim4 cell lines were provided by Gordon J. Freeman (Dana-Farber Cancer Institute, Boston, MA) and cultured in DMEM/F12 medium supplemented with 10% FBS, 10 mM HEPES (Gibco), 1% L-Glutamine and 1% Penicillin-Streptomycin. 1 µg/ml blasticidin was added to select for mouse Tim-4 transfectant 3T3 cells. For EV-depleted medium preparation, 20% FBS containing RPMI was ultracentrifuged for 16 hours at 100,000 *g* at 4 °C in a 45Ti fixed angle rotor using polycarbonate tubes (both from Beckman Coulter). After ultracentrifugation, the top 50 ml of medium suspension were harvested, filtered with 0.2 µm PES filter bottles and stored at 4 °C. Before using for culture, RPMI and L-glutamine, Penicillin-Streptomycin and ß-mercaptoethanol were added, to achieve the concentrations before mentioned. To produce DC2.4-derived EVs, cells were cultured for 2–3 days in EV-depleted medium and supernatants harvested before confluence was reached. Supernatants were first depleted of cells, debris and apoptotic bodies by serially centrifuging at 300 (10 min), 2000 (10 min) and 10,000 g (30 min) respectively, and EVs pelleted by ultracentrifugation at 100,000 *g* for 70 minutes at 4 °C in a 70Ti rotor (Beckman Coulter). This last step was repeated a second time in a 120.1 rotor (Beckman Coulter) and final EV pellet resuspended in PBS at ~10^11^ EV/ml and stored at 4 °C. EV depleted medium subjected to same EV isolation method was used as negative control and absence of particles was confirmed by NTA and nanoFACS (Fig. 2). HEK293 derived EVs were produced and isolated as described by Watson *et al*.^29^.

### Nanoparticle Tracking Analysis of EVs and polystyrene beads

Particle concentration and size distribution were characterized by NTA with a NanoSight LM10 instrument (Malvern), equipped with a 405 nm LM12 module and EM-CCD camera (DL-658-OEM-630, Andor). As an internal control across experiments and to ensure the accuracy of the analyses, we performed measurements of 100 nm NIST polystyrene beads (National Institute of Standards and Technology) immediately before each of the NTA the experiments. DC2.4-derived EVs were pre-diluted in PBS to achieve a concentration within the 10^8^–10^9^ range for optimal NTA analysis. The video acquisitions were performed with NTA software v3.1, using a 13–14 camera level for EVs and 10–11 for NIST polysytrene beads. 3–5 videos of 30 seconds were captured per sample. For the analyses, the threshold was set up at 5, and automatic blur size and 11.4–12.9 pixel maximum jump size selected. 100 and 200 nm polystyrene Yellow/Green bead (ThermoFisher) video captures were performed with camera level 12 and 9, respectively.

### EV labeling with lipid and protein-binding dyes

1–5 × 10^8^ EVs in PBS were stained by adding them on top of dye preparations as follows: for PKH26 staining (Sigma Aldrich), EVs diluted in 100 μl of diluent C (provided by manufacturer) were pipetted onto 100 μl of a 15 μM solution of PKH26 (final 7.5 μM PKH26), and incubated for 3 min at room temperature, as described before^16^. CM-DiI (Life Technologies) staining was performed likewise, but substituting diluent C for PBS. For 5-(and-6)-Carboxyfluorescein Diacetate Succinimidyl Ester (CFDA-SE, hereinafter CFSE) staining (ThermoFisher, catalog number V12883), the working solution was 200 µM in PBS, obtained by dilution from a previous 10 mM dye solution in DMSO. We avoided freeze and thaw cycles and exposure to light. For the staining, 15 μl of EV-containing PBS solution was pipetted onto 15 μl of a 40 μM CFSE solution, and incubated for 2 h at 37 °C, unless otherwise indicated. Tubes were mixed by flicking every hour. To analyze the formation of dye aggregates by nanoFACS or NTA, EV preparations were diluted up to 1 ml with PBS, without performing size exclusion chromatography. These experiments were repeated at least three times with similar results.

### CFSE unbound dye removal by size exclusion chromatography

To have enough sample for nanoFACS and NTA, we stained 8 samples of 2.5 × 10^9^ EVs each in 30 µl for 2 hours at 37 °C, as described previously. Then, we pooled them in two samples of 120 µl each and loaded them on two Illustra NAP-5 Size Exclusion Chromatography columns (GE Healthcare). We harvested 16 fractions of 250 μl each, following manufacturer’s instructions, and then pooled the fractions from the two columns to analyze each fraction simultaneously by nanoFACS and NTA. These experiments were repeated at least three times with similar results.

### NanoFACS analyses of EVs and beads

NanoFACS was performed with an Astrios EQ flow cytometer (Beckman Coulter), a jet-in-air system with 5 lasers (355, 405, 488, 561 and 640 nm wavelength), where SSC can be detected and used as a trigger in all laser paths, except for the 355 laser. We selected 561-SSC triggering and adjusted the 561-SSC voltage and threshold settings to allow 10,000–13,000 events of background reference noise per second. For fluorescence detection, we used a 513/26 band pass filter for CFSE, 579/16 for PKH26 and 614/20 for CM-DiI. The instrument was aligned using 200 nm polystyrene Yellow/Green beads and DC2.4 EVs. Samples were loaded and run for 10 minutes until the event rate was stable, and then 15 second acquisitions were saved. All samples were run at a 0.3 psi differential pressure, monitoring stability closely. Data were acquired using Summit v6 (Beckman Coulter) and analyzed with FlowJo v10.1r5 (TreeStar). Samples were run within a suitable operational range, according to Fig. 2F, without exceeding 100,000 events/second limit and therefore avoiding any coincident detection of particles or misleading results, unless otherwise indicated (Figs 2E–G and 3B). A decrease in background reference noise rate was used as an indicator of coincident detection of particles and the shift of background reference noise fluorescence indicated the presence of free dye in the sample preparation. Total event rate and background reference noise rate are routinely monitored to avoid misinterpretation of the results (such as coincident detection of particles) and to ensure good quality of the data.

### CFSE^+^ EV fluorescence and resolution limit quantification with MESF beads

FITC MESF beads were purchased from Bangs Laboratories and run according to manufacturer’s instructions, along with CFSE-stained EVs, without modifying the flow cytometer setup and voltages, except for FSC and SSC parameters. Blank and brightest bead were excluded from the analyses. Median fluorescence intensity (MedFI) values and FITC MESF values were transformed to logarithmic scale before doing the linear regression. EV MedFI values were also transformed to logarithmic scale to calculate their MESF value. MESF histogram was calculated likewise, exporting individual fluorescence intensity values for each EV or background reference noise event from FlowJo. The fluorescence resolution was calculated with the MedFI value of the events contained within a gate drawn on the edge of the background reference noise population, using a 2% counter plot in FlowJo. These experiments were repeated at least three times with similar results.

### Labeled EV uptake experiment

5 × 10^9^ EVs were labeled with CFSE as described before. As controls, we used CFSE dye alone and unstained EVs. Labeled EVs and controls were subjected to size exclusión chromatography as described before and 150 µl of the eluted fractions 3 and 4 (~10^9^ EVs), were used for the uptake experiment. DC2.4 cells were prestained with 0.2–0.5 µM of Cell Trace Far Red dye, following manufacturer’s instructions and resuspended in phenol red RPMI supplemented with 1% L-Glutamine. 100 µl containing two million pre-stained DC2.4 cells were incubated with 150 µl of EV or dye alone preparation at 37 °C for 6–12 h. Cells were washed twice before the analysis by microscopy or conventional flow cytometry. For confocal microscopy, 8 well chamber slides were used, pretreated with Poly-L-Lysine 0.01% (10 minutes at RT and subsequent washing with PBS). Experiments using 3T3 and 3T3-mTim4 cell lines were performed likewise but incubating the cells with HEK293 EVs^29^ for 3 hours. PKH26-labeled EVs or control dye alone were washed by airfuging for 30 minutes on top of a 30% sucrose cushion, using a A-95 rotor (Beckman), prior to use for cellular uptake experiments.

### Fluorescence Confocal Imaging

Images were collected on LSM780 confocal microscope (Carl Zeiss, Inc, Thornwood, NY) using 100X, 1.46 NA Zeiss objective and GaASP detector. During image acquisition cells were incubated in CO_2_/heating control stage insert at 37 °C with 5% CO_2_. Cell mid-sections were acquired at 12-bit image depth with line averaging (setting 4) and XY pixel size 83 nm (Fig. 5 and Supplementary Fig. 4) or 166 nm (Supplementary Fig. 5) in the following channels: Cell Trace Far Red dye (Ex 635 nm; Em 650–758 nm) and CFSE dye (Ex 488 nm; Em 490–553 nm) for Fig. 5 and Supplementary Fig. 5, or PKH26 (Ex 561 nm; Em 570–624 nm) for Supplementary Fig. 4. Images were scaled to 8-bit RGB identically in Zen software (Carl Zeiss, Inc, Thornwood, NY) and exported in JPEG format. Figures were made from those JPEG images in Power Point without any change in resolution. Scale bars are provided for each image.

### Data analyses and statistics

Mean, median and mode fluorescence intensities of gated populations and event counts of samples acquired by nanoFACS were analyzed using FlowJo (TreeStar). For ROC curves, median fluorescence intensity (MedFI) values of 10% of the total events in the EV gate (“patient values”) and background reference noise gate (“control values”) were plotted in GraphPad to get the area under the curve (AUC). The nonlinear regression analyses of event rate (Fig. 2) and linear regression for MESF beads were performed using GraphPad. Data acquired by NTA were analyzed with GraphPad to plot EV size and concentration depicting histograms or graphs (mean ± SD). The magnitude of separation between the EV fluorescence signal and background reference noise signal was calculated as follows: EV_MedFI_/Noise_MedFI_.

## Electronic supplementary material

Supplemental Figures

## References

[1] Colombo M, Raposo G, Thery C (2014). Biogenesis, secretion, and intercellular interactions of exosomes and other extracellular vesicles. Annu Rev Cell Dev Biol.

[2] Thery C, Ostrowski M, Segura E (2009). Membrane vesicles as conveyors of immune responses. Nat Rev Immunol.

[3] Melo SA (2015). Glypican-1 identifies cancer exosomes and detects early pancreatic cancer. Nature.

[4] Lasser, C., Eldh, M. & Lotvall, J. Isolation and characterization of RNA-containing exosomes. *J Vis Exp*, e3037, doi:10.3791/3037 (2012). 10.3791/3037 PMC3369768 22257828

[5] Morales-Kastresana, A. & Jones, J. C. In Exosomes and Microvesicles. Methods and Protocols Vol. 1545 (ed Andrew F. Hill) Ch. Flow Cytometric Analysis of Extracellular Vesicles, 215–225 (Springer, 2017). 10.1007/978-1-4939-6728-5_16 PMC7888554 27943218

[6] Arraud N (2014). Extracellular vesicles from blood plasma: determination of their morphology, size, phenotype and concentration. J Thromb Haemost.

[7] van der Pol E (2014). Particle size distribution of exosomes and microvesicles determined by transmission electron microscopy, flow cytometry, nanoparticle tracking analysis, and resistive pulse sensing. J Thromb Haemost.

[8] Akers JC (2016). Comparative Analysis of Technologies for Quantifying Extracellular Vesicles (EVs) in Clinical Cerebrospinal Fluids (CSF). PLoS One.

[9] Jeong S (2016). Integrated Magneto-Electrochemical Sensor for Exosome Analysis. ACS Nano.

[10] Wunsch BH (2016). Nanoscale lateral displacement arrays for the separation of exosomes and colloids down to 20 nm. Nat Nanotechnol.

[11] Im H (2014). Label-free detection and molecular profiling of exosomes with a nano-plasmonic sensor. Nat Biotechnol.

[12] Bobrie, A., Colombo, M., Krumeich, S., Raposo, G. & Thery, C. Diverse subpopulations of vesicles secreted by different intracellular mechanisms are present in exosome preparations obtained by differential ultracentrifugation. *J Extracell Vesicles* **1**, doi:10.3402/jev.v1i0.18397 (2012). 10.3402/jev.v1i0.18397 PMC3760636 24009879

[13] Stoner SA (2016). High sensitivity flow cytometry of membrane vesicles. Cytometry A.

[14] Groot Kormelink T (2016). Prerequisites for the analysis and sorting of extracellular vesicle subpopulations by high-resolution flow cytometry. Cytometry A.

[15] Hoen ENMN-'t (2012). Quantitative and qualitative flow cytometric analysis of nanosized cell-derived membrane vesicles. Nanomedicine.

[16] van der Vlist EJ, Nolte-‘t Hoen EN, Stoorvogel W, Arkesteijn GJ, Wauben MH (2012). Fluorescent labeling of nano-sized vesicles released by cells and subsequent quantitative and qualitative analysis by high-resolution flow cytometry. Nat Protoc.

[17] Arraud N, Gounou C, Turpin D, Brisson AR (2016). Fluorescence triggering: A general strategy for enumerating and phenotyping extracellular vesicles by flow cytometry. Cytometry A.

[18] Hoshino A (2015). Tumour exosome integrins determine organotropic metastasis. Nature.

[19] Peinado H (2012). Melanoma exosomes educate bone marrow progenitor cells toward a pro-metastatic phenotype through MET. Nat Med.

[20] Lyons, A. B., Blake, S. J. & Doherty, K. V. Flow cytometric analysis of cell division by dilution of CFSE and related dyes. *Curr Protoc Cytom* Chapter 9, Unit 9 11, doi:10.1002/0471142956.cy0911s64 (2013). 10.1002/0471142956.cy0911s64 23546777

[21] Perfetto SP, Ambrozak DR, Roederer M, Koup RA (2004). Viable infectious cell sorting in a BSL-3 facility. Methods Mol Biol.

[22] Bergsdorf C, Beyer C, Umansky V, Werr M, Sapp M (2003). Highly efficient transport of carboxyfluorescein diacetate succinimidyl ester into COS7 cells using human papillomavirus-like particles. FEBS Lett.

[23] Hoefel D, Grooby WL, Monis PT, Andrews S, Saint CP (2003). A comparative study of carboxyfluorescein diacetate and carboxyfluorescein diacetate succinimidyl ester as indicators of bacterial activity. J Microbiol Methods.

[24] Boing, A. N. *et al*. Single-step isolation of extracellular vesicles by size-exclusion chromatography. *J Extracell Vesicles* **3**, doi:10.3402/jev.v3.23430 (2014). 10.3402/jev.v3.23430 PMC4159761 25279113

[25] Hong CS, Funk S, Muller L, Boyiadzis M, Whiteside TL (2016). Isolation of biologically active and morphologically intact exosomes from plasma of patients with cancer. J Extracell Vesicles.

[26] Nordin JZ (2015). Ultrafiltration with size-exclusion liquid chromatography for high yield isolation of extracellular vesicles preserving intact biophysical and functional properties. Nanomedicine.

[27] Monguio-Tortajada M (2017). Nanosized UCMSC-derived extracellular vesicles but not conditioned medium exclusively inhibit the inflammatory response of stimulated T cells: implications for nanomedicine. Theranostics.

[28] Gamez-Valero A (2016). Size-Exclusion Chromatography-based isolation minimally alters Extracellular Vesicles’ characteristics compared to precipitating agents. Sci Rep.

[29] Watson DC (2016). Efficient production and enhanced tumor delivery of engineered extracellular vesicles. Biomaterials.

[30] Lai CP (2015). Visualization and tracking of tumour extracellular vesicle delivery and RNA translation using multiplexed reporters. Nat Commun.

[31] Thery, C., Amigorena, S., Raposo, G. & Clayton, A. Isolation and characterization of exosomes from cell culture supernatants and biological fluids. *Curr Protoc Cell Biol* Chapter 3, Unit 3 22, doi:10.1002/0471143030.cb0322s30 (2006). 10.1002/0471143030.cb0322s30 18228490

[32] Kobayashi N (2007). TIM-1 and TIM-4 glycoproteins bind phosphatidylserine and mediate uptake of apoptotic cells. Immunity.

[33] Kelleher RJ (2015). Extracellular Vesicles Present in Human Ovarian Tumor Microenvironments Induce a Phosphatidylserine-Dependent Arrest in the T-cell Signaling Cascade. Cancer Immunol Res.

[34] Witwer, K. W. *et al*. Standardization of sample collection, isolation and analysis methods in extracellular vesicle research. *J Extracell Vesicles* **2**, doi:10.3402/jev.v2i0.20360 (2013). 10.3402/jev.v2i0.20360 PMC3760646 24009894

