# Supplementary material for: Labeling Extracellular Vesicles for Nanoscale Flow Cytometry
Source: Sci Rep. 2017 May 12;7:1878. doi: 10.1038/s41598-017-01731-2 (PMC5431945; doi:10.1038/s41598-017-01731-2)
Supplement: Supplementary file 1 — Supplemental Figures [file 41598_2017_1731_MOESM1_ESM.pdf]

Supplementary information

Labeling Extracellular Vesicles for Nanoscale Flow Cytometry

Aizea Morales-Kastresana[1], Bill Telford[2], Thomas A Musich[3], Katherine McKinnon[4], Cassandra Clayborne[1], Zach Braig[1], Ari Rosner[1,2], Thorsten Demberg[3], Dionysios C Watson[5], Tatiana S. Karpova[6], Gordon J. Freeman[7], Rosemarie H. DeKruyff[8], George N Pavlakis[5], Masaki Terabe[1], Marjorie Robert-Guroff[3], Jay A Berzofsky[1], Jennifer C Jones[1*]

Supplementary Fig. 1

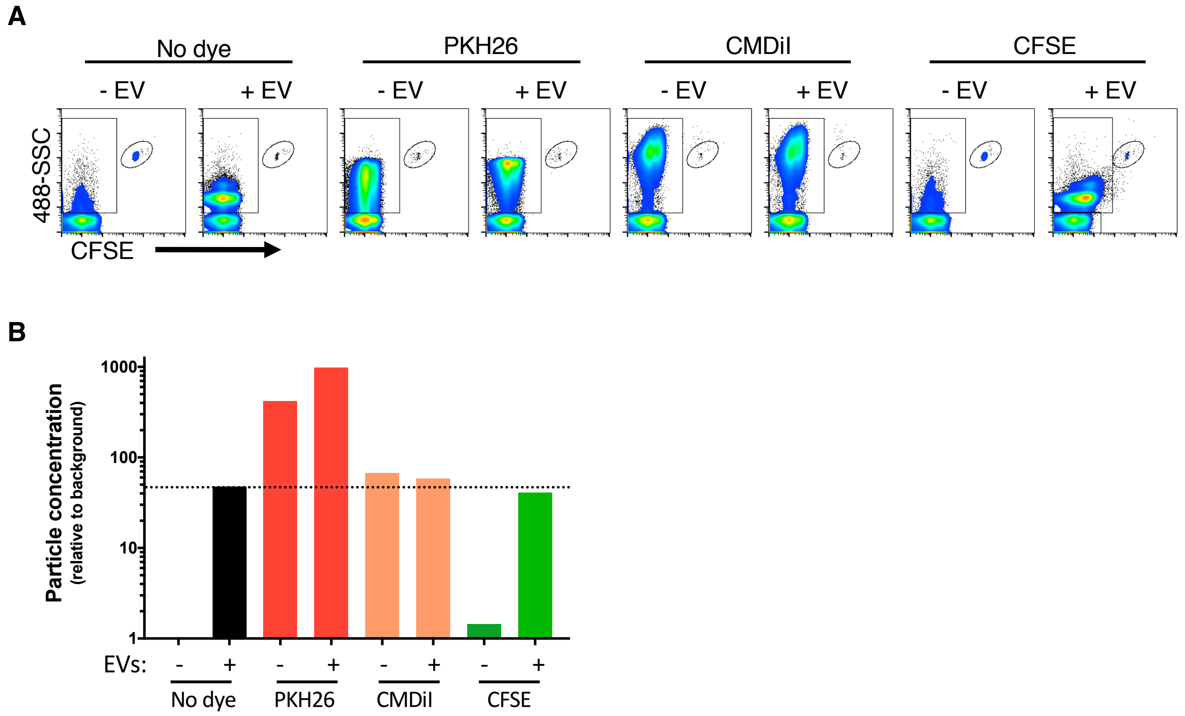

Supplementary Fig. 1. Quantitative nanoFACS analysis of EVs stained with lipophilic and protein-binding dyes. (**A**) Samples prepared as in **Fig. 3A-D** were spiked with 200 nm polystyrene beads at a known concentration. The concentration of particles in each sample was calculated based on the particle (rectangular gate) and bead counts (round gate) as described in the methods section. A small population of non-fluorescent events is always observed in sheath fluid or PBS, even with 40nm inline filtration, due to degassing nano-bubbles that form when the 60 PSI sheath fluid exits the nozzle in ambient pressure, before crossing the laser-stream intercept point. These events represent 2-4% of total events and do not interfere with EV analysis. (**B**) Particle concentration relative to background PBS (No dye and no EV). The dotted line represents the relative concentration of unstained EVs.

Supplementary Fig. 2

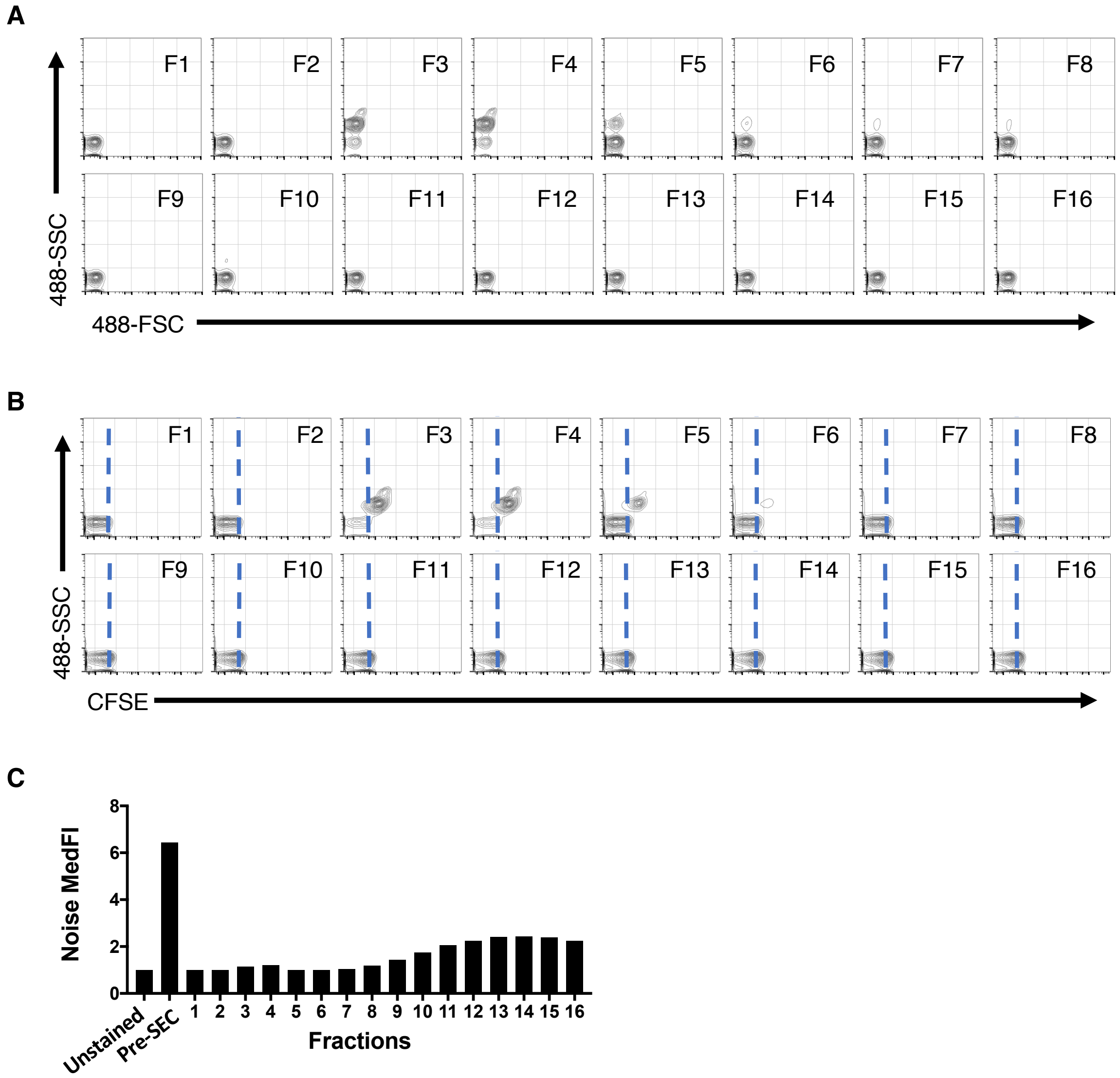

Supplementary Fig. 2. CFSE^{+} EV elution profile after size exclusion chromatography. (**A**) 488-FSC – 488-SSC and (**B**) CFSE fluorescence contour plots of EV fractions shown in **Fig. 4** that were collected after size exclusion chromatography. (**C**) MedFI of background reference noise as gated in **Fig. 2E**. These are representative plots of an experiment, repeated multiple times, with similar results. F, fraction; Pre-SEC, before size exclusion chromatography; MedFI, Median Fluorescence Instensity.

Supplementary Fig. 3

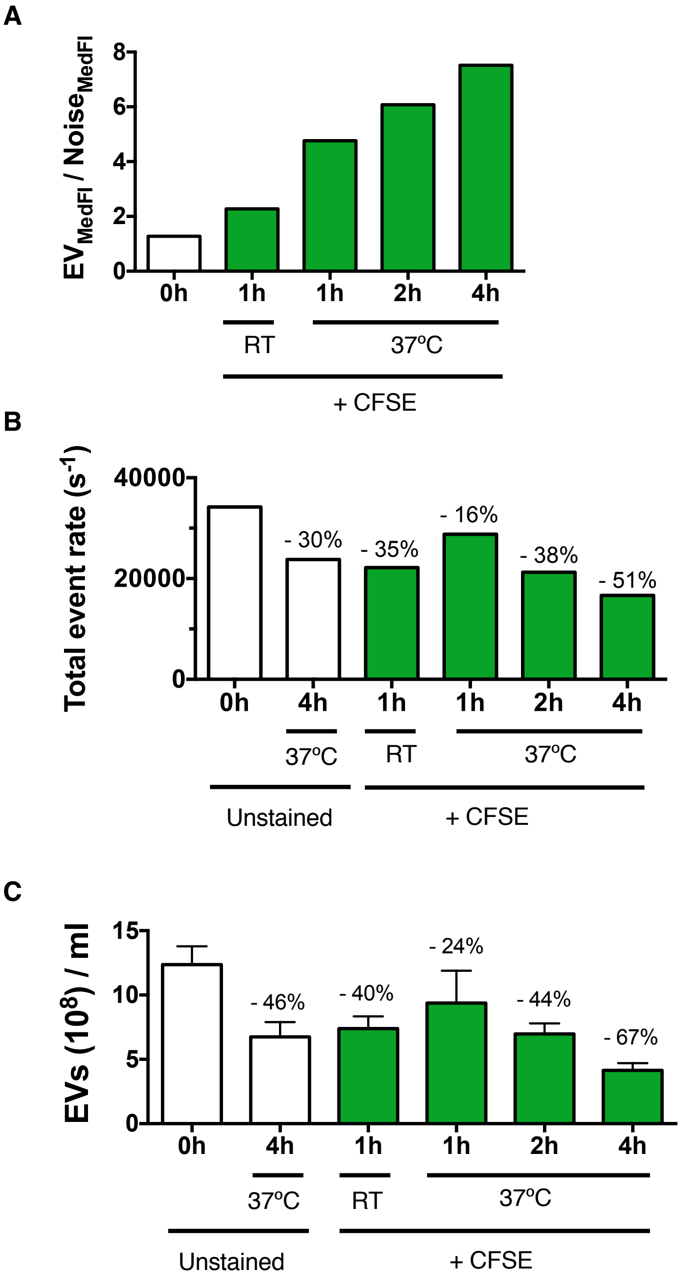

Supplementary Fig. 3. CFSE fluorescence increases and EV number decreases with higher temperature and longer incubation time.
(**A**) EV/noise MedFI ratio analysis of EVs incubated as indicated. All samples, including the unstained control, were washed with size exclusion chromatography columns. (**B**) Total event rate (s^{-1}) of samples shown in **A** and additional unstained EVs incubated for 4h at 37ºC. (**C**) EV concentration quantification by NTA of samples shown in **B**. Numbers on top of columns represent the decrease percentage compared to unstained EVs at time zero. Mean ± SD of five replicates is represented in **C**.

Supplementary Fig. 4

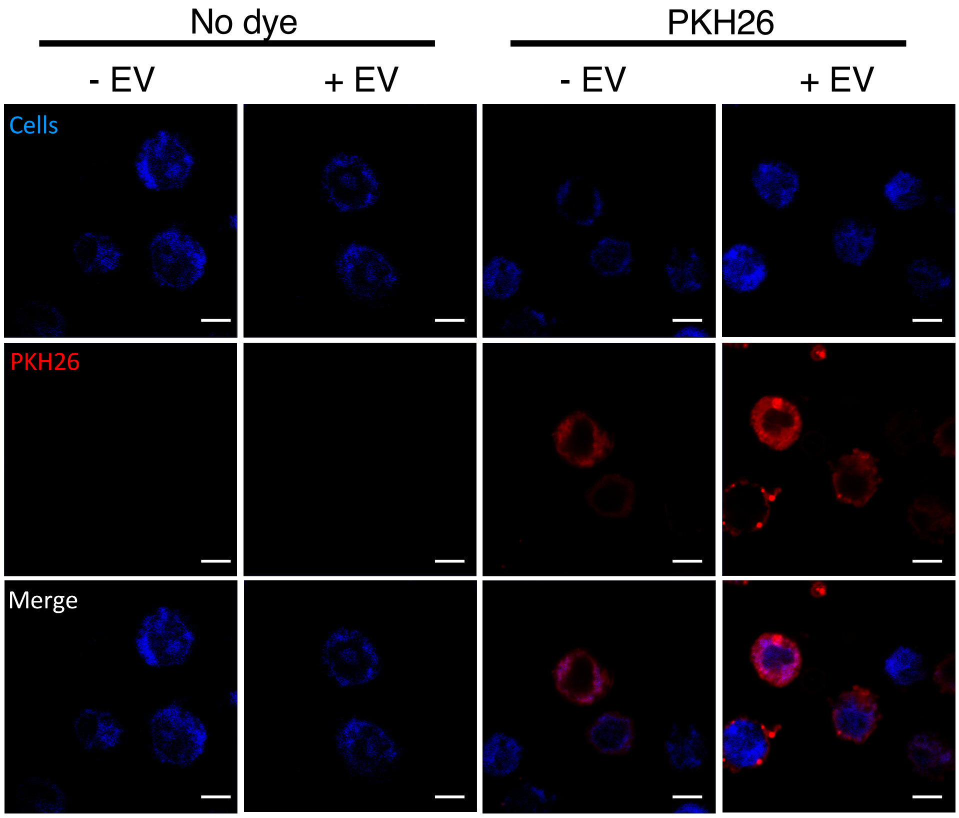

Supplementary Fig. 4. PKH26 dye micelles non-specifically stain cells. Similar experiment to **Fig. 5**, but instead using PKH26 as a label for EVs. DC2.4 cells incubated with unstained EVs remain negative for PKH26 signal, while cells incubated with PKH26-labeled EV or dye alone preparations become fluorescent, indicating uptake of EVs or direct labeling with PKH26 dye and micelles. Representative images out of 5 analyzed fields are shown. All images were scaled in the same way. The experiment was repeated twice with similar results. Scale bar = 5 μm.

Supplementary Fig. 5

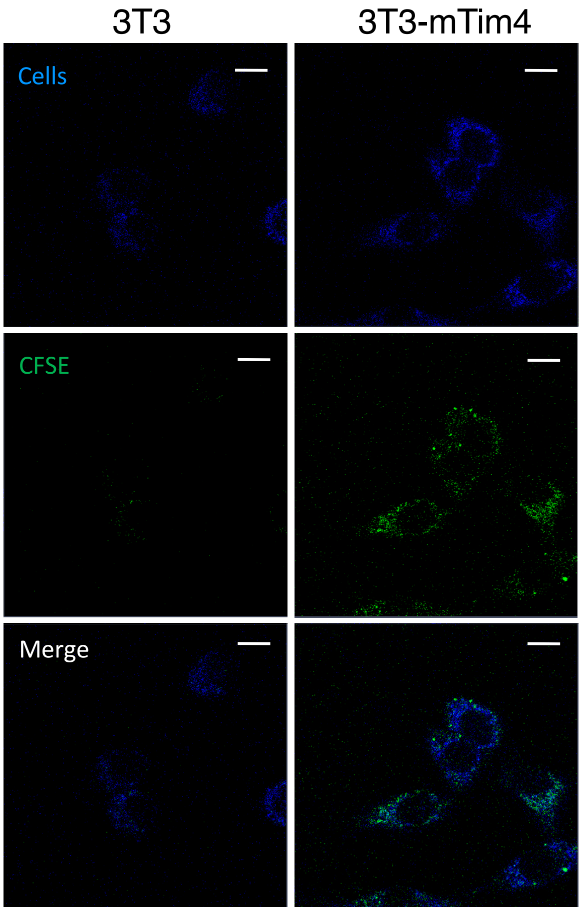

Supplementary Fig. 5. Molecular recognition of EV epitopes is preserved on CFSE-labeled EVs. EVs labeled with CFSE and cultured with untransfected (left column) or Tim4-transfected (right column) 3T3 lines showing an increase of EV uptake mediated by TIM-4. Cells stained with Cell Trace Far Red (in blue). Representative images out of 5 analyzed fields are shown. All images were scaled in the same way. Scale bar = 10 μm.

Supplementary Fig. 6

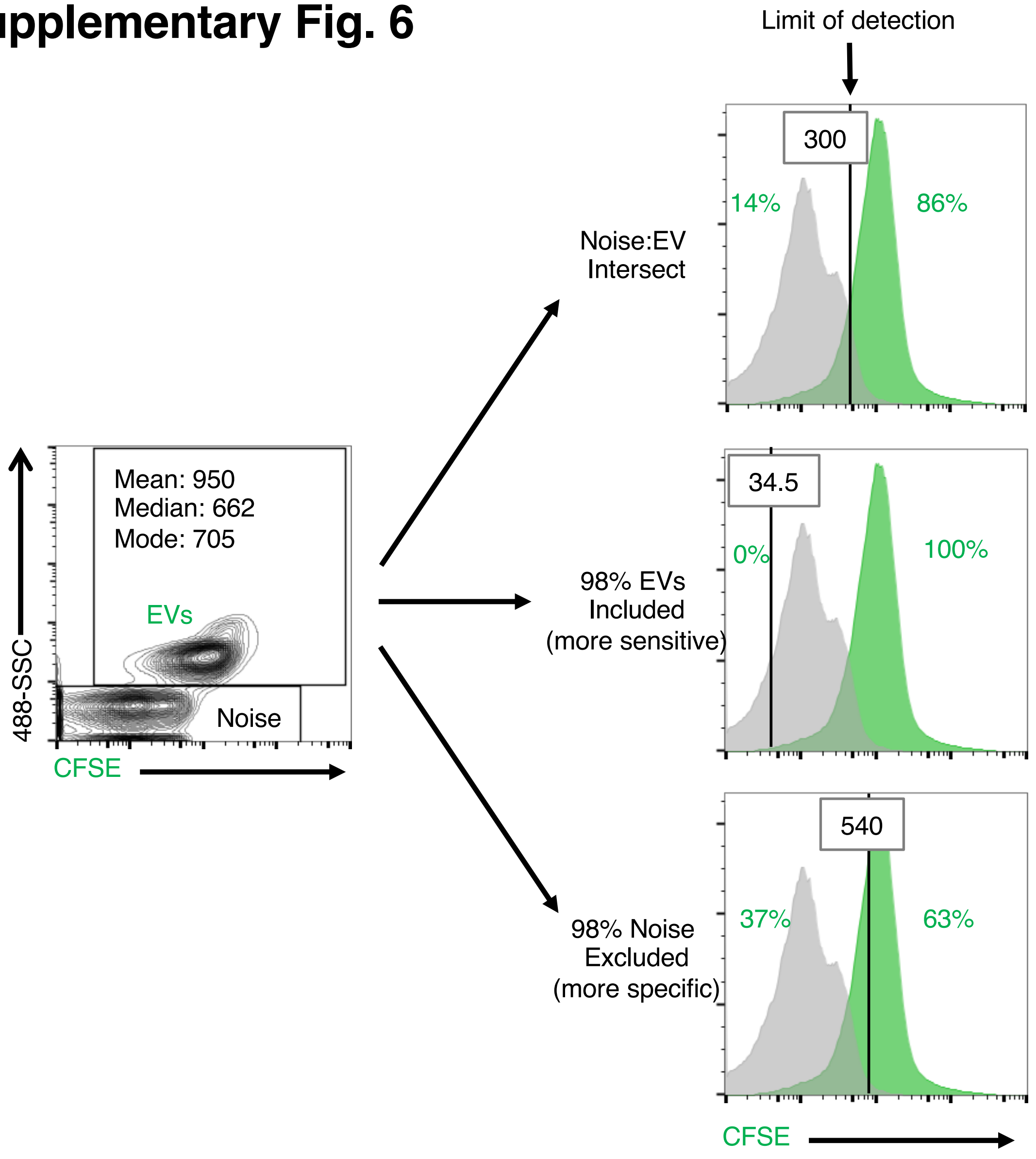

Supplementary Fig. 6. Despite excellent resolution of EVs with two dimensional analysis, the unidimensional limit of detection of CFSE-labeled EVs by nanoFACS depends on the sensitivity and specificity defined by the user.
EV and noise gating strategy and fluorescein MESF mean, median and mode values for EV gate are plotted on the left. On the right, overlaid histograms of noise (in gray) and EVs (in green) gated as indicated in the left contour plot. The vertical line represents the fluorescence detection threshold according to different criteria: EV and noise histogram intersect (top histogram), contains 98% of EVs (middle histogram) or excludes 98% of noise (lower histogram). The percentage of EVs (green) detected below and above the limit (left and right of each histogram, respectively) varies according to the position of the fluorescence threshold. The limit of detection, in MESF units, is depicted within the box. Experiment repeated three times with similar results.

Supplementary Fig. 7

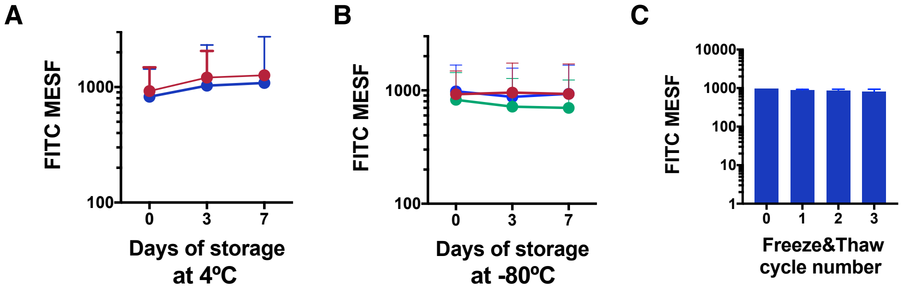

Supplementary Fig. 7. CFSE staining is stable within EVs at 4ºC and -80ºC.
(A and **B)** EVs stained with CFSE for 2h at 37ºC and washed by size exclusion chromatography were kept at 4ºC (**A**) or aliquots frozen at -80 (**B**) to analyze the MESF values by nanoFACS on the indicated days. Experiment was stopped after day 7 because EVs were no longer detected when samples were stored at 4ºC. Graphs show paired data of two (**A**) and three (**B**) independent experiments. Error bars correspond to rSD. (**C**) MESF values of CFSE-stained EVs that were subjected to repetitive freeze and thaw cycles. Mean ± SD of is shown. MESF, Molecular of Equivalent Soluble Fluorochrome; rSD, robust Standard Deviation.
